# A Narrative Review of Antibiotic Prescribing Practices and Antimicrobial Resistance Challenges in Conflict-Affected Sudan

**DOI:** 10.3390/antibiotics15060547

**Published:** 2026-05-29

**Authors:** Hamid Mn. Mustafa, Tahani Elfaki, Ishag Adam

**Affiliations:** 1 Department of Pharmacy Practice, College of Pharmacy, Qassim University, Buraydah 51452, Saudi Arabia; ha.mustafa@qu.edu.sa; 2 Clinical Pharmacology Department of Pharmacology, Faculty of Pharmacy, University of Gezira, Wad Madani 21111, Sudan; tahani2007@uofg.edu.sd; 3 Department of Obstetrics and Gynecology, College of Medicine, Qassim University, Buraydah 51452, Saudi Arabia

**Keywords:** antibiotic prescribing, antimicrobial use, antimicrobial resistance, antimicrobial stewardship, ambulatory care, outpatient care, community pharmacy, infection prevention and control, Sudan, conflict settings, AWaRe classification

## Abstract

**Background:** Antimicrobial resistance (AMR) is a growing global health threat, disproportionately affecting low- and middle-income countries such as Sudan. Conflict-related health system disruption has further intensified inappropriate antibiotic use and weakened stewardship capacity. **Objective:** This narrative review synthesizes contemporary evidence on antibiotic prescribing practices in Sudan, with emphasis on ambulatory care, and examines their implications for AMR control in the context of ongoing conflict. **Methods:** A non-systematic, structured narrative review was conducted successfully. PubMed, Google Scholar, WHO/EMRO databases, and Sudan’s National Action Plan (NAP) materials were examined for literature published between January 2010 and December 2025. Peer-reviewed research, government guidelines, surveillance reports, and gray literature were among the eligible sources. A total of 78 studies were included after titles, abstracts, and full texts were screened. Two reviewers independently confirmed the data extraction, and the synthesis aligned with SANRA guidelines. **Results:** Antibiotic prescribing in Sudan is frequently inappropriate, particularly in ambulatory and community settings, where empirical treatment, polypharmacy, and extensive use of Watch antibiotics are common. Alignment with WHO AWaRe recommendations remains suboptimal. Recent clinical and molecular evidence demonstrates increasing multidrug resistance among *Escherichia coli*, *Klebsiella pneumoniae*, *Pseudomonas aeruginosa*, and *Staphylococcus aureus*, including ESBL production and emerging carbapenemase genes (e.g., NDM 1, IMP 1). Conflict-related disruptions—such as reduced laboratory capacity, supply chain breakdown, and unregulated community dispensing—have further accelerated AMR. Pilot stewardship interventions show promise but remain limited in scale. **Conclusions:** Inappropriate antibiotic use in Sudan is driven largely by ambulatory and community practices and has been exacerbated by conflict. Strengthening stewardship beyond hospitals, enforcing prescription-only regulations, operationalizing the AWaRe framework, and aligning empirical therapy with local resistance patterns are critical for mitigating AMR in Sudan and similar conflict-affected settings.

## 1. Introduction

### 1.1. Global Overview of Antimicrobial Resistance

Antimicrobial resistance (AMR) is a major global public health challenge that threatens the effectiveness of antimicrobial agents used to treat bacterial, viral, fungal, and parasitic infections [1,2,3]. The emergence and spread of AMR are accelerated by inappropriate antibiotic prescribing, self-medication, incomplete treatment courses, and extensive antimicrobial use across human, animal, and environmental sectors [1,2,3]. AMR undermines routine medical procedures, increases morbidity and mortality, and imposes substantial economic burdens on health systems [4,5,6,7,8].

In response, the World Health Organization (WHO) launched the Global Action Plan on AMR in 2015, urging Member States to adopt multisectoral national action plans grounded in a One Health approach [4,9].

### 1.2. Sudan’s AMR Landscape and Antibiotic Use Patterns

Sudan faces a disproportionately high AMR burden due to structural fragility, weak regulatory enforcement, and widespread over-the-counter antibiotic availability [10,11]. Antibiotic consumption is high across public and private sectors, with extensive use of broad-spectrum agents and frequent empirical prescribing [12,13,14]. Community pharmacies frequently dispense antibiotics without prescriptions, contributing to inappropriate antibiotic exposure and undermining stewardship efforts [15,16,17].

Sudan launched its National Action Plan (NAP) on AMR in 2018 with support from WHO, FAO, and WOAH, emphasizing rational antimicrobial use, surveillance strengthening, and infection prevention and control [10,11]. However, implementation has been uneven, and stewardship activities remain limited outside tertiary hospitals. Table 1 summarizes the key national governance structures involved in AMR control in Sudan, including the Federal Ministry of Health, WHO partners, the TAP program, and the national laboratory network. These institutions collectively shape surveillance, stewardship, and policy implementation efforts.

### 1.3. Impact of Conflict on Antibiotic Use and AMR in Sudan

The armed conflict that erupted in April 2023 has profoundly disrupted Sudan’s health system. Key conflict-related disruptions include:

- Collapse of microbiology and diagnostic services;
- Disruption of supply chains for essential access to antibiotics;
- Expansion of informal antibiotic markets;
- Mass displacement and increased reliance on humanitarian care;
- Overcrowded shelters and weakened infection prevention and control;
- Loss of healthcare workers and stewardship oversight.

These disruptions have intensified empirical prescribing, increased self-medication, and weakened regulatory enforcement, accelerating AMR emergence [24,25,26,27,28].

### 1.4. Rationale for This Review

This review integrates prescribing practices, pathogen-specific resistance patterns, stewardship initiatives, and conflict-related health system disruptions—an approach not previously synthesized in the Sudanese context.

## 2. Results

### 2.1. Antimicrobial Resistance Patterns in Sudan and GLASS

#### 2.1.1. Antimicrobial Resistance Patterns in Sudan

As shown in Table 2, resistance patterns vary substantially across healthcare sectors, with ICUs exhibiting the highest burden of MDR organisms, while ambulatory settings show widespread resistance to first-line oral agents. Community-level resistance is strongly influenced by access to non-prescription antibiotics.

Recent evidence demonstrates that antimicrobial resistance (AMR) in Sudan has intensified across healthcare settings, with substantial variation by level of care, pathogen profile, and geographic region. Hospital-based studies consistently report high resistance to commonly used antibiotic classes—including β-lactams, third-generation cephalosporins, and fluoroquinolones, particularly among *Escherichia coli* and *Klebsiella pneumoniae* isolates [29,30,36]. The prevalence of extended-spectrum β-lactamase (ESBL)-producing *Enterobacterales* has increased markedly, often necessitating carbapenem use as a last-line therapeutic option [29,36].

In intensive care units (ICUs), resistance patterns are more severe. Studies report a high burden of multidrug-resistant (MDR) *Klebsiella* spp. and *Acinetobacter* spp., with carbapenem resistance frequently exceeding 50% in some centers [31]. Colistin remains one of the few reliably active agents, although emerging resistance has been noted. Appendix Table A1 summarizes resistance mechanisms among hospital and ICU pathogens, including carbapenemase-producing *Klebsiella*, MDR *Acinetobacter*, and XDR *Pseudomonas*.

Emergency and acute care settings show extensive empirical antibiotic use, often without microbiological confirmation. Several hospitals report elevated methicillin-resistant *Staphylococcus aureus* (MRSA) prevalence, driven by broad-spectrum prescribing, overcrowding, and delayed diagnostic support [32,37].

Ambulatory and outpatient settings exhibit similar reliance on empirical treatment. Resistance to first-line oral agents—including amoxicillin–clavulanate, ciprofloxacin, and cefixime—is increasingly documented. Aminoglycosides, particularly amikacin and gentamicin, remain among the most active agents against Gram-negative pathogens in these settings [31,36].

At the community level, rising AMR trends are closely linked to self-medication and non-prescription antibiotic dispensing, facilitated by weak regulatory enforcement and widespread community pharmacy access [15,16,38]. National surveillance data, including WHO GLASS reporting, indicate that MDR organisms account for approximately 30–40% of isolates, with Gram-negative bacteria predominating and substantial regional variation in resistance profiles [21,30,35].

Appendix Table A2 (refer to Appendix A) provides detailed resistance profiles for major community-acquired pathogens, including *E. coli*, *S. aureus*, and *K. pneumoniae*.

Burden estimates further highlight the severity of AMR in Sudan. In 2021, approximately 3940 deaths were directly attributable to bacterial AMR, with an additional 14,600 deaths associated with resistant infections. Sudan ranked 99th globally for age-standardized AMR mortality and was among the highest-burden countries in the Middle East and North Africa region [39].

Conflict-affected settings exacerbate AMR patterns due to disrupted healthcare delivery, limited laboratory capacity, medicine shortages, and unregulated antibiotic use. These conditions accelerate resistance development and undermine stewardship and surveillance efforts [25,28].

##### Pathogen-Specific Resistance Patterns in Sudan

*Pseudomonas aeruginosa*

*P. aeruginosa* demonstrates rapidly increasing MDR, XDR, and PDR phenotypes, particularly in hospital and ICU settings. Resistance increasingly affects carbapenems and antipseudomonal β-lactams [31,40]. Molecular studies have confirmed the presence of carbapenemase genes, including New Delhi Metallo β-lactamase 1 (NDM 1) and Imipenemase 1 (IMP 1), in clinical isolates from Khartoum, indicating advanced genetic drivers of resistance [41].

*Escherichia coli*

*E. coli*, the predominant uropathogen in Sudan, shows high resistance to first-line oral agents such as amoxicillin–clavulanate, fluoroquinolones, and cephalosporins. These patterns are largely attributed to widespread ESBL production, documented in both hospital and community settings [33,42,43].

*Staphylococcus aureus*

*S. aureus* displays a distinct resistance profile, with high resistance to amoxicillin–clavulanate and erythromycin. While susceptibility to vancomycin and linezolid remains largely preserved, emerging vancomycin intermediate and vancomycin-resistant strains have been reported in selected centers [32,33,42,44].

#### 2.1.2. Sudan’s Participation in AMR Surveillance and GLASS

Sudan is formally enrolled in the WHO Global Antimicrobial Resistance and Use Surveillance System (GLASS). However, reporting remains inconsistent due to limited laboratory capacity, supply chain disruptions, and conflict-related interruptions. The WHO GLASS Report 2025 highlights substantial gaps in isolate-based surveillance, standardized laboratory reporting, and submission of epidemiological metadata [21].

Irregular data submission and low completeness scores reflect long-standing system constraints, including shortages of trained microbiologists, limited access to culture and susceptibility testing, and fragmented reporting pathways. These challenges have been further exacerbated by the 2023 conflict, which has reduced laboratory functionality in several states and disrupted national coordination mechanisms [25,28].

### 2.2. Antibiotic Prescribing Patterns in Sudan

Evidence consistently demonstrates that antibiotic prescribing in Sudan is both high in volume and frequently inappropriate, particularly in ambulatory and community settings. Across multiple studies, common patterns include empirical prescribing, polypharmacy, limited adherence to treatment guidelines, and extensive use of broad-spectrum antibiotics.

#### 2.2.1. Pre-Conflict Prescribing Patterns (2010–2022)

Before the 2023 conflict, prescribing practices in Sudan were already characterized by:

- High antibiotic prescription rates in both public and private sectors [13,14].
- Frequent use of broad-spectrum agents, especially third-generation cephalosporins and fluoroquinolones [45,46,47,48].
- Limited diagnostic support, leading to reliance on empirical therapy [18,46].
- Polypharmacy, particularly in hospital settings [17,18].
- Poor adherence to national standard treatment guidelines [13,17,49].
- Widespread nonprescription dispensing in community pharmacies [15,16].

Public-sector facilities generally reported lower prescribing volumes but greater reliance on older broad-spectrum antibiotics due to restricted formularies and supply limitations [13,14]. Private-sector facilities showed higher prescribing rates, a stronger influence of patient demand, and more frequent use of Watch-category antibiotics [13,14,45].

Table 3 highlights key differences between public- and private-sector prescribing. Private facilities demonstrate higher prescribing frequency and greater reliance on Watch antibiotics, while public facilities are constrained by limited formularies but still show substantial empirical use.

#### 2.2.2. Post-Conflict Prescribing Patterns (2023–2025)

The conflict that began in April 2023 significantly worsened prescribing practices. Key changes include:

- Increased empirical prescribing due to the collapse of microbiology services and diagnostic shortages [25,28].
- Greater use of Watch antibiotics (e.g., ceftriaxone, ciprofloxacin) because Access antibiotics became scarce due to supply chain disruptions [25,28].
- Expansion of informal and unregulated antibiotic markets, including street vendors and cross-border drug flows [25].
- Higher rates of self-medication as access to formal healthcare declined [14,28,50].
- Reduced stewardship oversight due to the displacement of healthcare workers and loss of institutional governance [25,28].

These conflict-related disruptions have intensified inappropriate antibiotic use and accelerated AMR emergence.

#### 2.2.3. Alignment with WHO AWaRe Classification

Across both sectors, alignment with the WHO AWaRe framework remains suboptimal:

- Access to antibiotics (e.g., amoxicillin, doxycycline) is underutilized in many settings despite being recommended as first-line agents [46,47].
- Watch for antibiotics (e.g., ceftriaxone, ciprofloxacin, azithromycin) that are frequently prescribed empirically, even for mild or self-limiting infections [45,46,47].
- Reserve antibiotics are rarely used, but stewardship oversight is limited.

These patterns reflect gaps in guideline dissemination, prescriber training, and regulatory enforcement.

#### 2.2.4. Drivers of Inappropriate Prescribing

Multiple structural and behavioral factors contribute to irrational antibiotic use in Sudan:

- Limited diagnostic capacity and reliance on syndromic management [18,46].
- High expectations for antibiotics, especially in private clinics [13,14,45].
- Commercial incentives in private facilities and community pharmacies [14,45,50].
- Weak enforcement of prescription-only regulations [16,17].
- Inadequate stewardship infrastructure, particularly outside hospitals [14,17,49].

Collectively, these drivers create a prescribing environment where broad-spectrum and unnecessary antibiotic use are common.

### 2.3. Antibiotic Prescribing Across Healthcare Facilities and Stakeholder Groups in Sudan

Antibiotic prescribing practices in Sudan vary substantially across healthcare facilities and stakeholder groups, reflecting differences in diagnostic capacity, regulatory oversight, economic incentives, and patient expectations. Evidence from primary care, hospitals, community pharmacies, informal providers, and the general public demonstrates a complex ecosystem in which multiple actors contribute to inappropriate antibiotic use and the emergence of AMR.

#### 2.3.1. Primary Healthcare and Ambulatory Care Settings

Studies consistently show that primary healthcare centers (PHCCs) and ambulatory clinics have the highest antibiotic prescribing rates in Sudan. Antibiotics are frequently prescribed for self-limiting or viral infections, including upper respiratory tract infections, acute diarrhea, and nonspecific febrile illnesses [13,18,46,47].

Key patterns include:

- High empirical prescribing due to limited access to diagnostic tools [18,46].
- Frequent use of broad-spectrum agents, particularly ceftriaxone, azithromycin, and ciprofloxacin [45,46,47].
- Limited adherence to national treatment guidelines, especially for common infections [13,14,49].
- Short consultation times and high patient load, which encourage rapid empirical decisions [13,18].

Ambulatory care remains the primary driver of inappropriate antibiotic use in Sudan, given its large patient volume and minimal stewardship oversight.

#### 2.3.2. Hospitals and Secondary/Tertiary Care Facilities

Hospitals generally demonstrate more structured prescribing practices than primary care facilities, but inappropriate antibiotic use remains common. Contributing factors include:

- Incomplete implementation of hospital antibiotic policies [18,49].
- Limited routine audit and feedback mechanisms for prescribers [49].
- Variable stewardship capacity, with most programs concentrated in tertiary hospitals [49].
- High rates of polypharmacy, particularly in inpatient wards and surgical units [17,18].

While hospitals offer greater opportunities for stewardship interventions, the absence of standardized monitoring systems limits sustained improvements.

#### 2.3.3. Intensive Care Units (ICUs)

ICUs represent the highest-risk environment for AMR emergence due to:

- High use of broad-spectrum and last-line antibiotics;
- Critically ill patients with prolonged hospital stays;
- Frequent invasive procedures;
- Limited microbiology support in many facilities.

Studies report extremely high MDR rates among *Klebsiella* spp., *Acinetobacter* spp., and *Pseudomonas aeruginosa*, with carbapenem resistance frequently exceeding 50% [31]. Stewardship interventions in ICUs remain limited due to staffing shortages and diagnostic constraints.

#### 2.3.4. Community Pharmacies

Community pharmacies play a central role in access to antibiotics in Sudan. Multiple simulated patient studies and pharmacist surveys show:

- High rates of non-prescription antibiotic dispensing [15,16].
- Antibiotics supplied for viral infections, including colds and influenza-like illnesses [17,49].
- Dispensing decisions driven by patient demand, economic incentives, and perceived professional autonomy [16,17,49].
- Limited awareness or application of AMR and stewardship principles [16,17,49].
- Weak regulatory enforcement allows pharmacies to function as de facto primary care providers, significantly contributing to community-level antibiotic misuse.

#### 2.3.5. Informal Healthcare Providers

Informal providers, including traditional healers, unlicensed practitioners, and street vendors, are widely utilized, especially in rural and conflict-affected areas. Their practices are characterized by:

- Unregulated antibiotic supply, often without proper dosing or indication [17,49].
- Use of substandard or counterfeit antibiotics due to informal procurement channels [25].
- Minimal knowledge of AMR or treatment guidelines.

These providers represent a major but understudied source of inappropriate antibiotic exposure.

#### 2.3.6. The General Public

Public knowledge, attitudes, and practices significantly influence antibiotic use patterns. Studies show:

- Low awareness of AMR mechanisms and risks [13,16,17].
- Widespread belief that antibiotics are necessary for rapid symptom relief, regardless of etiology [13,16,51,52,53].
- High rates of self-medication, facilitated by easy access to antibiotics through pharmacies and informal markets [14,28,50].
- Limited exposure to structured AMR awareness campaigns.

These behavioral factors reinforce inappropriate prescribing and dispensing practices across the health system.

#### 2.3.7. Summary

Across all stakeholder groups, antibiotic use in Sudan is shaped by:

- Diagnostic limitations;
- Weak regulatory enforcement;
- Economic pressures;
- Public misconceptions;
- Limited stewardship capacity.

These interconnected factors create a prescribing environment that favors empirical, broad spectrum, and often unnecessary antibiotic use, contributing significantly to AMR emergence.

Table 4 synthesizes antibiotic use patterns across key stakeholder groups. Primary care settings show high empirical prescribing, community pharmacies frequently dispense antibiotics without prescriptions, and informal providers contribute to unregulated antibiotic access.

### 2.4. Knowledge and Attitudes Concerning Antibiotics and AMR Among Key Stakeholder Groups in Ambulatory Care in Sudan

Evidence from multiple knowledge, attitude, and practice (KAP) studies reveals persistent gaps in understanding, suboptimal attitudes, and inconsistent behaviors related to antibiotic use and antimicrobial resistance (AMR) among key stakeholder groups involved in ambulatory care in Sudan. These groups include physicians, community pharmacists, pharmacy technicians, and the general public. Although general awareness of AMR as a public health threat is relatively high, detailed knowledge of appropriate antibiotic selection, dosing, treatment duration, and stewardship principles remains limited and unevenly distributed across professions [16,17].

To address the reviewers’ recommendations, the findings are organized into five thematic domains.

#### 2.4.1. Prescriber Misconceptions and Clinical Decision-Making Challenges

Healthcare professionals working in ambulatory and outpatient settings often demonstrate moderate conceptual knowledge of AMR but limited ability to translate this knowledge into evidence-based prescribing decisions. Common misconceptions include:

- Belief that antibiotics are necessary for most febrile illnesses;
- Overestimation of bacterial infection prevalence in primary care;
- Underestimation of the risks associated with broad-spectrum antibiotic use.

These misconceptions are compounded by limited familiarity with national treatment guidelines and inconsistent awareness of the WHO AWaRe classification, particularly outside tertiary hospitals [13,15,18,49,50].

#### 2.4.2. Diagnostic Uncertainty and Structural Barriers

Diagnostic limitations are a major driver of inappropriate prescribing. In many primary care settings:

- Microbiological testing is unavailable;
- Rapid diagnostic tools are limited;
- Clinical decisions rely heavily on syndromic management.

This diagnostic uncertainty encourages empirical prescribing, often with broad-spectrum agents, even when clinical suspicion of bacterial infection is low [18]. The absence of routine culture and sensitivity testing also limits prescribers’ ability to align therapy with local resistance patterns.

#### 2.4.3. Economic and System-Level Drivers

Economic pressures and structural constraints significantly influence antibiotic use behaviors:

- In private clinics, patient expectations and commercial incentives encourage liberal prescribing [13,14,45].
- In community pharmacies, profit motives and customer retention concerns drive nonprescription antibiotic dispensing [16,17].
- In public facilities, medicine shortages and restricted formularies lead to reliance on whatever antibiotics are available, often older broad-spectrum agents [13].

These system-level drivers reinforce patterns of inappropriate antibiotic use across both formal and informal sectors.

#### 2.4.4. Public Behavioral Factors and Misconceptions

Studies consistently show that the general public in Sudan has low awareness of AMR mechanisms, limited understanding of appropriate antibiotic use, and widespread misconceptions, including:

- Belief that antibiotics accelerate recovery from any illness;
- Perception that injectable antibiotics are more effective;
- Reliance on leftover antibiotics or advice from non-medical acquaintances;
- Self-medication is common and facilitated by easy access to antibiotics without prescriptions through community pharmacies and informal providers [14,16,17,28]. These behaviors significantly contribute to community-level antibiotic misuse.

#### 2.4.5. Barriers to Engagement in Antimicrobial Stewardship

Across stakeholder groups, attitudes toward antimicrobial stewardship programs (ASPs) are generally positive. Healthcare workers recognize the potential of ASPs to improve patient outcomes and reduce AMR. However, several barriers limit effective engagement:

- Lack of formal training in stewardship principles;
- Limited institutional support and absence of dedicated stewardship teams;
- High workload and time constraints;
- Inadequate access to updated guidelines and local antibiograms.

These barriers are particularly pronounced in primary care and community settings, where stewardship activities remain minimal [22,25,50].

#### 2.4.6. Summary

Overall, KAP studies highlight a complex interplay of knowledge gaps, diagnostic limitations, economic pressures, and public misconceptions that collectively drive inappropriate antibiotic use in Sudan’s ambulatory care sector. Addressing these challenges requires integrated, multisectoral interventions that simultaneously target prescribers, dispensers, and the public.

Table 5 summarizes KAP findings across stakeholder groups, showing moderate AMR awareness among healthcare workers, persistent misconceptions, limited familiarity with guidelines, and widespread acceptance of non-prescription antibiotic dispensing.

### 2.5. Quality Indicators Currently Used in Ambulatory Care in Sudan

Assessment of antibiotic prescribing quality in ambulatory care settings in Sudan relies on a limited but gradually expanding set of indicators adapted from the WHO core drug use indicators and internationally recognized antimicrobial stewardship (AMS) metrics. Most evaluations focus on prescribing patterns, antibiotic selection, and adherence to treatment guidelines rather than patient-level outcomes or resistance trends.

#### 2.5.1. Prescribing Frequency and Appropriateness Indicators

Across multiple studies, the most consistently reported indicator is the high frequency of antibiotic prescribing, often exceeding WHO-recommended thresholds for primary care. Antibiotics are frequently prescribed for viral or self-limiting conditions, reflecting diagnostic uncertainty and prescriber misconceptions [13,18,46].

Indicators commonly assessed include:

- Percentage of encounters with an antibiotic prescribed;
- Percentage of antibiotics prescribed from the national essential medicines list;
- Percentage of broad-spectrum vs. narrow-spectrum antibiotics;
- Percentage of prescriptions adhering to national guidelines.

Findings consistently show overuse of broad-spectrum agents, particularly third-generation cephalosporins and fluoroquinolones, even for uncomplicated infections [49,54].

#### 2.5.2. AWaRe Classification Indicators

Several studies have begun evaluating antibiotic use through the WHO AWaRe framework, which categorizes antibiotics into Access, Watch, and Reserve groups. Key findings include:

- Underutilization of access to antibiotics, despite their recommendation as first-line agents [46,49,55].
- High reliance on Watch antibiotics (e.g., ceftriaxone, ciprofloxacin, azithromycin) in ambulatory care settings [45,47,49].
- Minimal use of Reserve antibiotics, although stewardship oversight remains limited.

The overuse of Watch antibiotics is one of the most important quality concerns in Sudan’s ambulatory sector, as it directly contributes to the selection of resistant pathogens.

#### 2.5.3. Diagnostic and Laboratory Indicators

Diagnostic capacity is a critical determinant of prescribing quality. Indicators commonly reported include:

- Availability of microbiology services;
- Percentage of prescriptions supported by laboratory testing;
- Turnaround time for culture and sensitivity results.

Most ambulatory settings lack access to microbiological diagnostics, resulting in near-universal empirical prescribing [18,46]. This limitation undermines adherence to guidelines and contributes to inappropriate antibiotic selection.

#### 2.5.4. Regulatory and Dispensing Indicators

Weak enforcement of prescription-only regulations is a major barrier to the quality use of antibiotics. Indicators used to assess regulatory compliance include:

- Percentage of antibiotics dispensed without a prescription;
- Pharmacy adherence to dispensing regulations;
- Availability of standard treatment guidelines in pharmacies.

Studies consistently show high rates of non-prescription antibiotic dispensing, particularly in community pharmacies, which undermines rational prescribing efforts [16,17].

#### 2.5.5. Data Collection and Monitoring Indicators

Data collection systems in ambulatory care remain fragmented. Most studies rely on:

- Prescription audits;
- Simulated patient assessments;
- Cross-sectional surveys;
- Manual record reviews.

Routine electronic monitoring systems are rare, and longitudinal quality indicators—such as trends in prescribing appropriateness or resistance-linked outcomes—are largely absent.

#### 2.5.6. Summary

Overall, quality indicators used in Sudan’s ambulatory care sector highlight persistent challenges, including:

- High antibiotic prescribing frequency;
- Overuse of Watch antibiotics;
- Limited diagnostic support;
- Weak regulatory enforcement;
- Fragmented monitoring systems.

Strengthening quality assessment frameworks—particularly through AWaRe-based monitoring, improved diagnostics, and regulatory oversight—is essential for improving antibiotic use and mitigating AMR in Sudan.

## 3. Discussion

This narrative review synthesizes evidence on antibiotic prescribing practices, antimicrobial resistance (AMR) patterns, and stewardship efforts in Sudan, with particular emphasis on ambulatory care and the impact of conflict-related health-system disruption. The findings reveal a complex interplay of structural, behavioral, and contextual factors that collectively drive inappropriate antibiotic use and accelerate AMR emergence.

### 3.1. Inappropriate Antibiotic Use as a Systemic Challenge

Across healthcare settings, antibiotic use in Sudan is characterized by high prescribing frequency, extensive empirical treatment, and reliance on broad-spectrum agents. These patterns are consistent with findings from other low- and middle-income countries (LMICs), where limited diagnostic capacity and weak regulatory enforcement contribute to irrational antibiotic use. However, Sudan’s situation is further complicated by longstanding health-system fragility and widespread availability of antibiotics without prescription.

Ambulatory care settings—where most patient encounters occur—represent the primary source of inappropriate antibiotic exposure. High patient expectations, short consultation times, and limited access to diagnostic tools reinforce empirical prescribing, often with antibiotics in the Watch category. These findings align with studies from Yemen, Nigeria, and Pakistan, where similar pressures drive overuse of broad-spectrum agents in primary care.

### 3.2. AMR Trends Reflect Diagnostic Gaps and Treatment Pressures

The review highlights increasing resistance among key pathogens, including *Escherichia coli*, *Klebsiella pneumoniae*, *Pseudomonas aeruginosa*, and *Staphylococcus aureus*. The rise in ESBL-producing Enterobacterales and carbapenemase-producing organisms mirrors trends observed in conflict-affected settings such as Syria, Iraq, and South Sudan [26,56]. Limited microbiology capacity in Sudan restricts culture-guided therapy, contributing to the delayed detection of resistant strains and reinforcing reliance on broad-spectrum antibiotics.

ICUs show the highest burden of multidrug-resistant organisms, reflecting global patterns in critical care environments. However, the emergence of carbapenemase genes such as NDM-1 and IMP-1 in Sudanese isolates underscores the urgency of strengthening laboratory surveillance and stewardship interventions.

### 3.3. Conflict-Related Disruptions Intensify AMR Risks

The 2023 conflict has profoundly disrupted Sudan’s health system, exacerbating pre-existing vulnerabilities. Supply chain breakdowns have reduced the availability of Access antibiotics, increasing reliance on Watch agents. The collapse of microbiology services has eliminated culture-guided prescribing in many regions, while the displacement of healthcare workers has weakened stewardship oversight.

These disruptions parallel experiences in Yemen and Syria, where conflict has led to increased empirical prescribing, expansion of informal drug markets, and accelerated AMR emergence. Sudan’s situation is further complicated by the proliferation of unregulated antibiotic vendors and the absence of functional regulatory enforcement in many areas.

### 3.4. Stewardship Efforts Show Promise but Remain Limited

Pilot antimicrobial stewardship (AMS) interventions—such as the Tailoring Antimicrobial Resistance (TAP) program—demonstrate that context-adapted, behavior-focused strategies can improve prescribing practices in primary care. However, stewardship activities remain concentrated in tertiary hospitals, with limited reach into ambulatory and community settings where most antibiotic use occurs.

Strengthening stewardship requires:

- Expanding AMS programs beyond hospitals;
- Integrating AWaRe-based prescribing tools;
- Enhancing diagnostic capacity;
- Improving regulatory enforcement;
- Implementing community-level awareness campaigns.

These priorities align with global recommendations for AMR control in fragile and conflict-affected settings.

### 3.5. Implications for Policy and Practice

The findings underscore the need for a multisectoral approach to AMR mitigation in Sudan, incorporating:

- Health-system strengthening to restore diagnostic services and supply chains;
- Regulatory reforms to curb non-prescription antibiotic dispensing;
- Training and capacity building for prescribers and pharmacists;
- Community engagement to address misconceptions and self-medication;
- Enhanced surveillance through GLASS and national laboratory networks.

Given the ongoing conflict, interventions must be adaptable, scalable, and feasible in resource-constrained environments.

## 4. Limitations

This narrative review has several limitations. First, the evidence base is heterogeneous, with studies varying in design, quality, and geographic coverage. Many data sources are cross-sectional and facility-based, limiting generalizability. Second, the review relies on published literature and available gray sources, which may introduce publication bias. Third, conflict-related disruptions have reduced the availability of recent surveillance data, particularly from rural and insecure regions. Fourth, although the review followed SANRA guidelines, the absence of a systematic search protocol limits reproducibility. Despite these limitations, the review provides a comprehensive synthesis of current evidence and highlights critical gaps requiring further investigation.

## 5. Materials and Methods

### 5.1. Overall Approach

This study employed a structured, non-systematic narrative review methodology, guided by the Scale for the Assessment of Narrative Review Articles (SANRA). The review aimed to synthesize evidence on antibiotic prescribing practices, AMR patterns, and stewardship initiatives in Sudan, with emphasis on ambulatory care and conflict-related disruptions.

### 5.2. Search Strategy

A comprehensive search was conducted in PubMed, Google Scholar, WHO and EMRO databases, and Sudan’s National Action Plan (NAP) documents. Searches covered the period from January 2010 to December 2025, with final retrieval on 15 January 2026 (some new searches were conducted). Search terms included combinations of: “antibiotic prescribing,” “antimicrobial resistance,” “Sudan,” “ambulatory care,” “community pharmacies,” “stewardship,” “conflict,” “ESBL,” “carbapenem resistance,” and “AWaRe.”

### 5.3. Eligibility Criteria

Included sources were:

- Peer-reviewed studies;
- National guidelines and policy documents;
- Surveillance reports (including GLASS);
- Gray literature relevant to AMR and antibiotic use in Sudan.

Studies were included if they reported data on prescribing practices, AMR patterns, stewardship interventions, or stakeholder knowledge.

### 5.4. Study Selection and Screening Process

Titles and abstracts were screened for relevance. Full texts of potentially eligible studies were reviewed. Duplicate records were removed. A total of 78 studies met the inclusion criteria.

### 5.5. Data Extraction and Verification

Data were extracted into structured matrices covering study characteristics, prescribing patterns, AMR findings, and stewardship activities. Extraction was independently verified by two reviewers, with discrepancies resolved through consensus.

### 5.6. Synthesis Approach

Findings were synthesized thematically across major domains: AMR patterns, prescribing practices, stakeholder knowledge, stewardship initiatives, and conflict-related disruptions. The synthesis prioritized clarity, coherence, and alignment with SANRA recommendations.

## 6. Conclusions

Antibiotic use in Sudan is characterized by high prescribing frequency, extensive empirical treatment, and widespread reliance on broad-spectrum agents, particularly in ambulatory and community settings. These patterns, combined with weak regulatory enforcement and limited diagnostic capacity, have accelerated the emergence of AMR. Conflict-related disruptions have further intensified inappropriate antibiotic use and undermined stewardship and surveillance systems.

Strengthening AMR control in Sudan requires coordinated action across sectors, including restoring diagnostic services, expanding stewardship programs beyond hospitals, enforcing prescription-only regulations, and implementing community-level awareness initiatives. Operationalizing the AWaRe framework and aligning empirical therapy with local resistance patterns are essential for preserving antibiotic effectiveness and supporting health-system recovery in conflict-affected settings.

## Figures and Tables

**Table 1:** This is a table showing national governance structures and ongoing institutional initiatives addressing antibiotic use and AMR in Sudan.

Group or Body	Key Activities/Achievements	References
Federal Ministry of Health (FMoH), Sudan	Endorsed National AMR Action Plan (2018); issued national antibiotic prescribing guidelines (2016); oversaw stewardship and surveillance	[11,18]
WHO (Sudan Country Office & EMRO)	Technical support for NAP, stewardship, and surveillance; enabled GLASS enrolment and reporting	[18,19]
TAP programme (FMoH–WHO)	Theory-based behavior-change intervention in primary care (education, training, modeling, persuasion); targeted prescribers and patients; pilot in Gezira State	[18]
Primary health-care facilities (Gezira State)	Implemented TAP pilot in 8 PHCCs; participation of prescribers and patients; context-specific interventions	[18]
National AMR surveillance system/GLASS focal points	Routine AST reporting; gradual expansion of surveillance coverage; contribution to regional and global AMR estimates	[20,21]
National laboratory network (incl. NPHL)	Conducts AST for priority pathogens; reports bloodstream, urinary, GI, and urogenital infections; improves data completeness	[22]
One Health partners (Animal Resources, Agriculture; WHO–FAO–WOAH)	Multisectoral AMR governance; antimicrobial use addressed across human, animal, and environmental sectors	[11,23]
Public awareness initiatives (e.g., WAAW)	National campaigns to promote rational antibiotic use and AMR awareness	[10]

AMR, antimicrobial resistance; AST, antimicrobial susceptibility testing; EMRO, World Health Organization Eastern Mediterranean Regional Office; FAO, Food and Agriculture Organization; FMoH, Federal Ministry of Health (Sudan); GI, gastrointestinal; GLASS, Global Antimicrobial Resistance and Use Surveillance System; NAP, National Action Plan (on AMR); NPHL, National Public Health Laboratory; PHCCs, primary health-care centers; TAP, Tailoring Antimicrobial Resistance program; WAAW, World Antibiotic Awareness Week; WHO, World Health Organization; WOAH, World Organisation for Animal Health (formerly OIE).

**Table 2:** Summary of antimicrobial resistance patterns across healthcare sectors in Sudan.

Sector/Setting	Key Findings	References
Hospital/Inpatient	High resistance to β-lactams, cephalosporins, and fluoroquinolones; ESBL-producing *E. coli* is common; carbapenems are often last-line	[29,30]
Intensive Care Units (ICUs)	High MDR *Klebsiella* and *Acinetobacter*; carbapenem resistance frequently >50%; colistin remains active	[31]
Emergency/Acute Care	Broad empirical use; elevated MRSA prevalence in some hospitals; limited lab-guided therapy	[29,32]
Ambulatory/Outpatient	High resistance to first-line oral agents; amikacin and gentamicin remain the most active	[33,34]
Community	Rising resistance linked to self-medication and non-prescription dispensing	[25,30]
National/Multi-site	MDR prevalence 30–40%; Gram-negative dominance; strong regional variation	[21,35]
Conflict-Affected Settings	Conflict worsens AMR via disrupted diagnostics, supply shortages, and unregulated antibiotic use	[25,28]

**Table 3:** Comparison of antibiotic utilization patterns in public and private healthcare sectors in Sudan.

Aspect	Public Sector Facilities	Private Sector Facilities	References
Overall Antibiotic Prescribing	High but constrained by availability	Very high prescribing frequency	[13,14,45,50]
Polypharmacy	Common, especially in hospitals	More frequent in ambulatory care	[17,18]
Guideline Adherence	Variable; often limited	Generally poor	[13,14,17,49]
AWaRe Alignment	Predominantly Access antibiotics	Increased use of Watch antibiotics	[46,47]
Drivers of Prescribing	Limited diagnostics; high workload	Patient demand; profit incentives	[13,14,45,50]

**Table 4:** Antibiotic utilization patterns across healthcare facilities and stakeholder groups.

Setting/Stakeholder	Key Prescribing or Use Patterns	AMR-Relevant Concerns	References
Primary and Ambulatory Care	High empirical prescribing	Overuse; poor diagnostic support	[13,18,46,47]
Hospitals	Variable prescribing practices	Weak stewardship enforcement	[18,49]
Community Pharmacies	Non-prescription dispensing common	Regulatory gaps; inappropriate use	[15,16]
Informal Providers	Unregulated antibiotic supply	High misuse risk	[17]
General Public	Self-medication; low AMR awareness	Increased resistance selection	[14,28,45,50]

**Table 5:** Knowledge, attitudes, and perceptions regarding antibiotics and AMR among stakeholder groups.

Stakeholder Group	Knowledge Level	Attitudes & Perceptions	Key Gaps & Challenges	References
Physicians (Ambulatory Care)	Moderate AMR awareness; variable guideline familiarity	Recognize AMR risk but rely on empirical prescribing	Limited diagnostics; low AWaRe awareness	[13,15,18,49,50]
Community Pharmacists	Moderate AMR awareness	Acceptance of non-prescription dispensing	Patient pressure; weak regulation	[16,17]
Pharmacy Technicians	Variable and often limited	Dispensing guided by availability	Lack of formal AMR training	[16]
General Public	Low AMR awareness	Antibiotics are perceived as a fast relief	Self-medication; poor health literacy	[16,17]
Healthcare Workers (Overall)	Conceptual AMR knowledge	Positive views on ASPs	Limited resources and support	[22,25,50]

## Data Availability

No new data were created or analyzed in this study. Data sharing is not applicable to this article.

## Abbreviations

The following abbreviations are used in this manuscript:

AMR	Antimicrobial Resistance
AMS	Antimicrobial Stewardship
AST	Antimicrobial Susceptibility Testing
AWaRe	Access, Watch, Reserve (WHO Antibiotic Classification)
β lactams/B lactams	Beta lactam Antibiotics
EMRO	World Health Organization Eastern Mediterranean Regional Office
ESBL	Extended Spectrum Beta Lactamase
FAO	Food and Agriculture Organization
FMoH	Federal Ministry of Health (Sudan)
GI	Gastrointestinal
GLASS	Global Antimicrobial Resistance and Use Surveillance System
HCWs	Healthcare Workers
ICU	Intensive Care Unit
IMP-1	Imipenemase 1
KAP	Knowledge, Attitudes, and Practices
LMICs	Low- and Middle-Income Countries
MDR	Multidrug Resistant/Multidrug Resistance
MRSA	Methicillin Resistant Staphylococcus aureus
MSF	Médecins Sans Frontières
NAP	National Action Plan (on AMR)
NDM 1	New Delhi Metallo β lactamase 1
NGOs	Non-Governmental Organizations
NPHL	National Public Health Laboratory
OCHA	United Nations Office for the Coordination of Humanitarian Affairs
PDR	Pan Drug Resistant
PHCCs	Primary Health Care Centers
TAP	Tailoring Antimicrobial Resistance Program
UTI	Urinary Tract Infection
WAAW	World Antibiotic Awareness Week
WHO	World Health Organization
WOAH (formerly OIE)	World Organisation for Animal Health (formerly Office International des Epizooties)
XDR	Extensively Drug Resistant

## Appendix A

**Table A1:** Pathogen-specific antimicrobial resistance mechanisms and implications for empirical therapy in Sudan.

Pathogen	Key Resistance Phenotypes	Documented Resistance Mechanisms	Main Clinical Settings	Implications for Empirical Therapy	Key References
*Pseudomonas aeruginosa*	MDR, XDR, PDR	Carbapenemase production (NDM-1, IMP-1); resistance to antipseudomonal β-lactams and fluoroquinolones	ICU, tertiary hospitals	Empirical carbapenem use increasingly unreliable; need for local antibiograms and reserve agents	[15,37,39]
*Escherichia coli*	High resistance to oral first-line agents	ESBL production; fluoroquinolone resistance	Community, outpatient, hospitals	Failure of amoxicillin–clavulanate and oral cephalosporins; Access agents often ineffective	[32,39,40]
*Staphylococcus aureus*	Lower MDR prevalence; MRSA present	Macrolide resistance; emerging vancomycin resistance	Hospitals, wounds and bloodstream infections	Vancomycin and linezolid are largely retained but require stewardship vigilance	[30,41]

MDR, multidrug resistant; XDR, extensively drug resistant; PDR, pandrug resistant; ESBL, extended-spectrum β-lactamase; ICU, intensive care unit.

**Table A2:** Hospital and ICU Resistant Pathogens and Key Resistance Mechanisms in Sudan.

Pathogen	Setting	Key Resistance Mechanisms	Most Active Agents	References
*Klebsiella pneumoniae*	ICU/Inpatient	Carbapenem resistance > 50%; ESBL; MDR	Colistin, tigecycline	[31]
*Acinetobacter baumannii*	ICU	MDR/XDR; carbapenem resistance; biofilm formation	Colistin	[31]
*Pseudomonas aeruginosa*	ICU/Surgical wards	Carbapenemase genes (NDM-1, IMP-1); MDR/XDR	Piperacillin-tazobactam (variable), colistin	[40,41]
*Escherichia coli*	Inpatient	ESBL; fluoroquinolone resistance	Amikacin, carbapenems	[29,42]
